# Decoy Receptor 3 Promotes Preosteoclast Cell Death via Reactive Oxygen Species-Induced Fas Ligand Expression and the IL-1*α*/IL-1 Receptor Antagonist Pathway

**DOI:** 10.1155/2020/1237281

**Published:** 2020-06-10

**Authors:** Yi-Jen Peng, Ching-Tsung Peng, Yi-Hsuan Lin, Gu-Jiun Lin, Shing-Hwa Huang, Shyi-Jou Chen, Huey-Kang Sytwu, Chia-Pi Cheng

**Affiliations:** ^1^Department of Pathology, Tri-Service General Hospital, National Defense Medical Center, Taipei, Taiwan; ^2^Department of Clinical Pharmacy, Armed Forces Taoyuan General Hospital, Taoyuan, Taiwan; ^3^Department and Graduate Institute of Biology and Anatomy, National Defense Medical Center, Taipei, Taiwan; ^4^Department of General Surgery, Tri-Service General Hospital, National Defense Medical Center, Taipei, Taiwan; ^5^Department and Graduate Institute of Microbiology and Immunology, National Defense Medical Center, Taipei, Taiwan; ^6^National Health Research Institutes, Miaoli, Taiwan

## Abstract

**Purpose:**

Interleukin-1*α* (IL-1*α*) is a potent cytokine that plays a role in inflammatory arthritis and bone loss. Decoy receptor 3 (DCR3) is an immune modulator of monocytes and macrophages. The aim of this study was to investigate the mechanism of DCR3 in IL-1*α*-induced osteoclastogenesis.

**Methods:**

We treated murine macrophages with DCR3 during receptor activator of nuclear factor kappa Β ligand- (RANKL-) plus IL-1*α*-induced osteoclastogenesis to monitor osteoclast formation by tartrate-resistant acid phosphatase (TRAP) staining. Osteoclast activity was assessed using a pit formation assay. The mechanisms of inhibition were studied by biochemical analyses, including RT-PCR, immunofluorescent staining, flow cytometry, an apoptosis assay, immunoblotting, and ELISA.

**Results:**

DCR3 suppresses IL-1*α*-induced osteoclastogenesis in both primary murine bone marrow-derived macrophages (BMM) and RAW264.7 cells as it inhibits bone resorption. DCR3 induces RANKL-treated osteoclast precursor cells to express IL-1*α*, secretory IL-1ra (sIL-1ra), intracellular IL-1ra (icIL-1ra), reactive oxygen species (ROS), and Fas ligand and to activate IL-1*α*-induced interleukin-1 receptor-associated kinase 4 (IRAK4). The suppression of DCR3 during RANKL- or IL-1*α*-induced osteoclastogenesis may be due to the abundant secretion of IL-1ra, accumulation of ROS, and expression of Fas ligand in apoptotic osteoclast precursor cells.

**Conclusions:**

We concluded that there is an inhibitory effect of DCR3 on osteoclastogenesis via ROS accumulation and ROS-induced Fas ligand, IL-1*α*, and IL-1ra expression. Our results suggested that the upregulation of DCR3 in preosteoclasts might be a therapeutic target in inflammatory IL-1*α*-induced bone resorption.

## 1. Introduction

Osteoclasts are differentiated from bone marrow-derived monocytes by stimulation of a critical factor, RANKL [1]. RANKL activation of its receptor RANK transduces downstream signals by recruiting TNF receptor-associated factors (TRAFs) [2]. This event triggers downstream signalling by stimulating its receptor RANK activating the nuclear factor *κ*B (NF-*κ*B) and mitogen-activated protein kinases (MAPKs) [3, 4]. The intracellular reactive oxidative stress (ROS) was also critically involved in RANKL/RANK signalling during osteoclastogenesis [5, 6]. IL-1 is known to be a potent cytokine in inflammatory regions, leading to bone erosion by activating osteoclasts [7, 8]. Overexpression of IL-1*α* in transgenic mice spontaneously induces polyarthritis [9, 10]. Furthermore, IL-1*α* has a synergistic effect in RANKL-stimulated osteoclastogenesis, which mediates TNF-*α* expression by directly stimulating the differentiation of osteoclast precursors and inducing RANKL overexpression in stromal cells [11].

DCR3 is a soluble protein that belongs to the tumour necrosis factor receptor superfamily [12]. DCR3 interacts with its ligands, including TNFSF6 (FASLG), TNFSF14 (LIGHT), and TNFSF15 (TL1A) [13–15]. The function of DCR3 is to block or compete with ligand-receptor downstream signalling. Previous studies have shown that DCR3 plays multiple roles in the immune system. DCR3 prevents heart allograft rejection [16], promotes cancer cell growth by escaping immune surveillance [17, 18], and ameliorates many animal models of autoimmune diseases [19–22]. Researchers have also found that DCR3 can modulate macrophage and dendritic cell differentiation and maturation [23, 24]. Our previous studies found that DCR3 global expression attenuates the disease severity of collagen-induced arthritis in a mouse model and suppresses osteoclast differentiation in vitro [25, 26]. Moreover, DCR3 has been reported to activate IL-1ra expression in tumour-associated macrophages [27]. A previous study has reported that IL-1*α* and IL-1ra counterregulate each other in murine keratinocytes [28]. These findings give us a hint that DCR3 might be involved in IL-1*α* and IL-1ra regulation.

In the present study, we assessed the effects of DCR3 on RANKL- plus IL-1*α*-induced osteoclastogenesis in RAW264.7 cells and murine bone marrow-derived macrophage (BMM) cells. Moreover, we evaluated the possible mechanisms of DCR3 in osteolytic inflammation based on RANKL-induced osteoclastogenesis.

## 2. Materials and Methods

### 2.1. Cell Line and Reagents

The RAW264.7 cell line was obtained from the Food Industry Research and Development Institute (FIRDI) in Taiwan. Recombinant human DCR3 was purchased from R&D Systems Inc. (Minneapolis, MN, USA). Recombinant mouse RANKL, M-CSF, IL-1*α*, and anti-IL-1*α* were purchased from PeproTech (London, UK). Anti-IL-1ra was purchased from Abcam (Cambridge, UK). All other reagents were purchased from Sigma-Aldrich (St. Louis, MO, USA).

### 2.2. Cell Culture of Murine RAW264.7

The murine monocyte/macrophage cell line RAW264.7 was cultured with DMEM (Gibco, Dublin, Ireland) containing 10% heat-inactivated FBS, penicillin (100 U/ml), and streptomycin (100 *μ*g/ml). All cells were grown in a humidified atmosphere containing 5% CO_2_ at 37°C. To induce osteoclast differentiation, RAW264.7 cells were suspended in *α*-MEM containing 10% FBS, 100 U/ml penicillin, and 100 *μ*g/ml streptomycin. Cells were seeded at a density of 1.5 × 10^4^ cells/ml in each kind of plate (1 ml/well for a 24-well plate; 200 *μ*l/well for a 96-well plate) and were stimulated with 50 ng/ml soluble RANKL alone or RANKL plus 50 ng/ml IL-1*α* concurrently treated with 10 *μ*g/ml DCR3 or IgG control for 5 days. The medium was replaced on day 3. The safety dosage of 10 *μ*g/ml DCR3 was used in RAW264.7 and BMM cells according to the previous studies by the cell viability assay [26, 29].

### 2.3. Cells Isolation and Osteoclast Differentiation

Bone marrow cells (BMMs) from normal DBA/1J mice tibia and femur bones were cultured overnight in *α*-MEM (Gibco BRL) supplemented with 10% heat-inactivated FBS, penicillin (100 U/ml), and streptomycin (100 *μ*g/ml). Nonadherent cells were harvested and cultured in the presence of 30 ng/ml M-CSF, 50 ng/ml RANKL, and 50 ng/ml IL-1*α* as well as 10 *μ*g/ml DCR3 or IgG for 5 days to generate osteoclasts. Cells were seeded at a density of 1 × 10^5^ cells/ml in each kind of plate (1 ml/well for a 24-well plate; 200 *μ*l/well for a 96-well plate). The medium was replaced on day 3.

### 2.4. Tartrate-Resistant Acid Phosphatase (TRAP) Staining

The cells were washed with PBS and fixed with 3.7% formaldehyde for 30 minutes. After washing with PBS, the cells were incubated at 37°C in a humid and light-protected incubator for 1 hour in the reaction mixture of a Leukocyte Acid Phosphatase Assay Kit (Cat. 387, Sigma-Aldrich) as directed by the manufacturer. The cells were washed three times with distilled water, and TRAP-positive multinucleated cells containing five or more nuclei were counted under a light microscope and photographed.

### 2.5. Pit Formation Assay

RAW264.7 cells were seeded onto 20 mm^2^ dentine slices (Cat. 3988, Corning) in 24-well plates at a density of 10^5^ cells per well. All the cultures were incubated in triplicate, and the cells were replenished every 3 days with fresh medium containing test articles. Then, the dentine slices were treated with 1 N NH_4_OH with sonication for 5 minutes. Resorption pits on the dentine slices were visualized by staining with Mayer's Hematoxylin Solution (Sigma-Aldrich). The ratios of the resorbed area to the total area were measured in four optical fields on a slice using NIH Image software at 100-fold magnification.

### 2.6. RT-PCR and QPCR Analysis

Total RNA was isolated from the cultured cells using the TRIzol Reagent (Invitrogen, Carlsbad, CA, USA) and reverse transcribed using SuperScript III Reverse Transcriptase (Invitrogen). PCR was performed using mouse-specific primers as shown in sTable 1. Thermal cycling parameters were 95°C for 5 minutes, followed by 25~35 cycles for 30 seconds at 95°C, 30 seconds at 61°C, 1 minute at 72°C, and 10 minutes at 72°C for the final elongation. The number of cycles for each gene was determined to be in the range of linear amplification through an optimization experiment. PCR products were separated on 1.2% agarose gels, visualized by ethidium bromide staining, and analyzed densitometrically using a phosphorimager and Quantity One software. The optical densities for each gene were normalized to the corresponding values for glyceraldehyde-3-phosphate dehydrogenase (GAPDH). All markers of QPCR were analyzed by the LightCycler 480 II system (Roche, Mannheim, Germany). Quantitative thermal cycling parameters were 95°C for 15 minutes, followed by 40 cycles for 30 seconds at 95°C, 30 seconds at 61°C, and 1 minute at 72°C, as well as 10 minutes at 72°C for extension. The relative levels of each values were evaluated and normalized with GAPDH.

### 2.7. Immunoblotting Analysis

Whole cell extracts were prepared according to our previous study [30]. In brief, RAW264.7 cells treated with DCR3 or IgG control in the absence or presence of RANKL or IL-1*α* were harvested and suspended in RIPA lysis buffer containing protease and phosphatase inhibitors. Equivalent amounts of protein (20 *μ*g/lane) were loaded into 10-15% SDS-PAGE for electrophoresis and transferred onto a polyvinylidene fluoride (PVDF) membrane. The membranes were blocked with 5% nonfat milk in PBST at room temperature for 1 hour twice to prevent nonspecific staining. Immunoblotting was performed using specific antibodies for IRAK4 (No. 4363), phospho-IRAK4 (No. 11927, Cell Signaling Technology, Danvers, MA, USA), IL-1*α* (No. 500-P51A stock concentration, 100 *μ*g/ml, PeproTech, London, UK), IL-1ra (No. ab124962 stock concentration, 61 *μ*g/ml, Abcam, Cambridge, UK), and GAPDH (No. 10494-1-AP stock concentration, 50 *μ*g/150 *μ*l, Proteintech Group, Rosemont, IL, USA). The membranes were incubated with primary antibodies at a diluted ratio of 1 : 1000 in room temperature for 1 hour. After washing three times in PBST for 10 mins, goat anti-rabbit secondary antibodies were added at diluted ratio of 1 : 2000 in room temperature for 1 hour. The immunoreactive bands were visualized with the enhanced chemiluminescent kit and analyzed by the VisionWorks LS UVP System. Each protein was measured by at least 3 times of repeated western blot analysis. The data were presented as the relative ratio of the target protein to the reference protein.

### 2.8. Immunofluorescent Staining

The distribution of IL-*1α* protein was assessed according to previously published protocols [31]. The cells were washed in PBS, fixed in 4% paraformaldehyde, permeabilized with 0.1% Triton X-100, incubated with 5% BSA, and then incubated with primary anti-IL-1*α* polyclonal antibody (1 : 50) at 4°C overnight. After overnight incubation, the cells were washed in PBS twice and incubated with secondary Alexa Fluor 488-conjugated donkey anti-rabbit IgG antibody for 2 hours (BioLegend, San Diego, CA, USA). After immunostaining, the cells were counterstained with the endoplasmic reticulum ER-ID® Red assay kit (Enzo Life Sciences, Farmingdale, NY, USA). Fluorescence was visualized using a Leica DMi8 fluorescence microscope at 40x magnification equipped with filters (A for Hoechst, GFP-EN for Alexa Fluor 488, and N21 for ER Texas Red) and analyzed by LAS EZ software.

### 2.9. Apoptosis Assays and Flow Cytometry

Cells were plated at a density of 10^5^ cells per well in 24-well plates under the protocol of osteoclast differentiation. After 48 hours of incubation, the cells were stained with Annexin V-FITC and PI for evaluating cell apoptosis or stained with PE-conjugated anti-Fas ligand to evaluate death ligand expression by the BD FACSCalibur flow cytometry system equipped with fluorescence detectors and bandpass filters, 530 nm for FITC and 585 nm for PE/PI (BD Biosciences, NJ, USA). Cells were acquired and analyzed by using CellQuest Pro software.

### 2.10. ROS Assays

Cells were plated at a density of 10^5^ cells per well in 24-well plates under the protocol of osteoclast differentiation. After 6 hours incubation, the CellROX Green Reagent (Invitrogen) was added to each well at a concentration of 5 *μ*mol/l according to the manufacturer. After 30 minutes of incubation, cells were analyzed by flow cytometry (BD Biosciences, NJ, USA) with 530 nm bandpass filters for green fluorescence and CellQuest Pro software.

### 2.11. Enzyme-Linked Immunosorbent Assay

Whole cell lysate and culture medium of IL-1*α* and IL-1ra were measured using ELISAs according to the manufacturer (murine IL-1*α* from eBioscience and IL-1ra from R&D Systems). Briefly, equivalent amounts of total cell lysate (5 *μ*g/well) or medium (100 *μ*l/well) were loaded into the IL-1*α* and IL-1ra specific antibody-precoated well and incubated overnight at 4°C. After washing with 200 *μ*l PBST 3 times, 100 *μ*l of diluted detection antibody was added to each well and incubated at room temperature for 1 hour. After 3 washes, 100 *μ*l Avidin-HRP was added to the wells and incubated at room temperature for 30 mins. After 5 washes, 100 *μ*l of 1x TMB solution was added to the wells and incubated at room temperature for 15 mins. The intensity of the color was measured at an absorbance wavelength of 450 nm. The detection limits were 4 pg/ml for IL-1*α* and 13 pg/ml for IL-1ra.

### 2.12. Statistical Analysis

All the experiments were done for at least 3 independent repeats. Data were shown as the mean values ± SD and were analyzed using one-way ANOVA with the Newman-Keuls multiple comparisons on posttests. *P* < 0.05 was considered statistically significant.

## 3. Results

### 3.1. Effects of DCR3 on RANKL- Plus IL-1*α*-Induced Osteoclast Differentiation and Function

**Figure 1:**
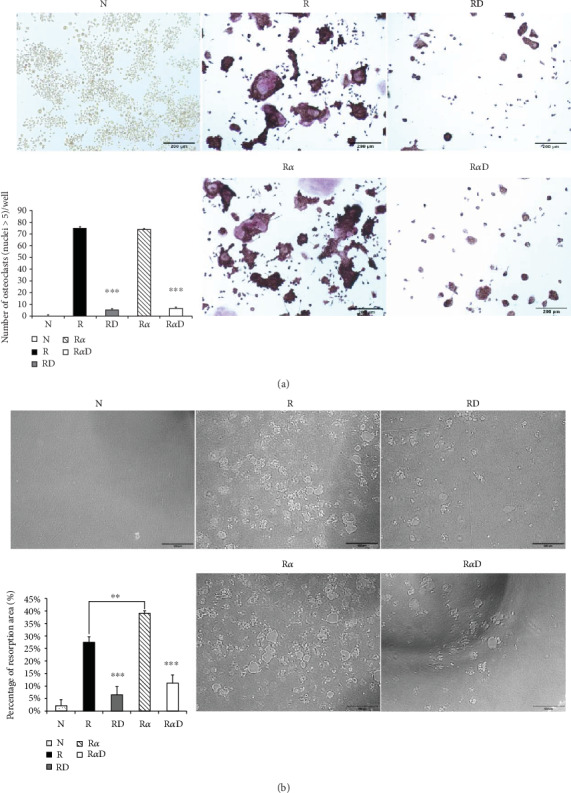
Effects of DCR3 on RANKL- plus IL-1*α*-induced osteoclast differentiation and function. (a) RAW264.7 cells were treated with DCR3 (10 *μ*g/ml) in the presence of RANKL (50 ng/ml) or RANKL plus IL-1*α* (50 ng/ml) for 5 days. After incubation, the cells were fixed and stained for TRAP, and TRAP^+^ multinucleated RAW264.7 cells containing more than five nuclei were counted as multinucleated osteoclasts. (b) RAW264.7 cells were seeded on dentine slices as described in the Materials and Methods. After being incubated for 5 days, the dentine slices were recovered from the culture and were subjected to a pit formation assay to visualize resorption. The percentages of the resorbed areas were determined using the NIH Image software. Data are presented as means ± SD of more than four slices and means ± SD of more than three cultures (N: RAW264.7 cells; R: RANKL; RD: RANKL+DCR3; R*α*: RANKL+IL-1*α*; R*α*D: RANKL+IL-1*α*+DCR3; ^∗∗^*P* < 0.01 and ^∗∗∗^*P* < 0.001).

Investigating the suppressive effect of DCR3 in IL-1*α*-induced osteoclastogenesis was promising. RANKL-stimulated RAW264.7 cells were treated with DCR3 in the presence or absence of IL-1*α*. The results showed that the numbers of multinucleated osteoclasts decreased in the DCR3-treated group even when they underwent concurrent treatment with IL-1*α* (Figure 1(a)). In BMMs, the IL-1*α*-treated group was more dramatically increased in the RANKL-induced osteoclast formation as compared with RANKL treated alone. And, these suppressive effects of DCR3 were also reproduced by using primary murine BMMs (sFigure 1). To further examine the effects of DCR3 on the functions of osteoclastogenesis, we evaluated bone resorption activity by using a pit formation assay. The results showed that RANKL- plus IL-1*α*-evoked bone resorption was diminished in the presence of DCR3 (Figure 1(b)).

### 3.2. Effects of DCR3 on IL-1*α* and IL-1ra Regulation in RANKL-Induced Osteoclast Differentiation

**Figure 2:**
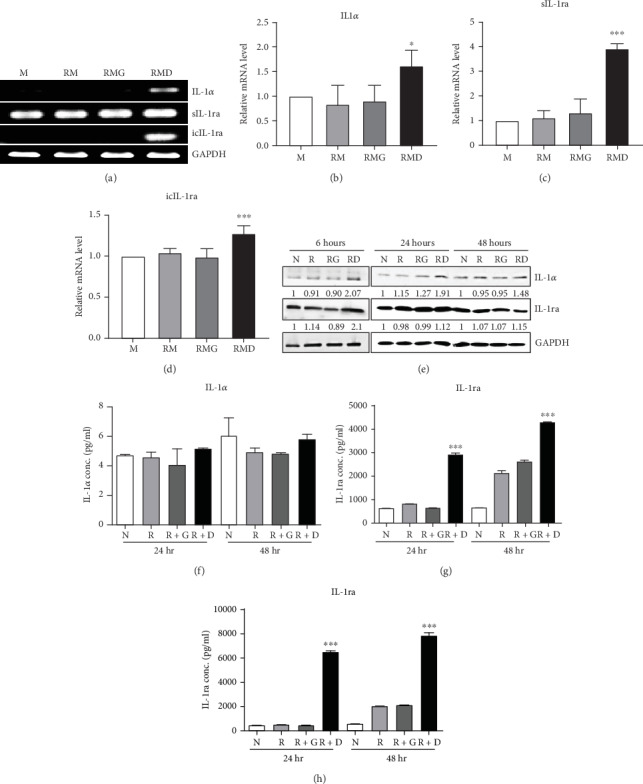
Effects of DCR3 on IL-1*α* and IL-1ra regulation on RANKL-induced osteoclast differentiation. (a–d) BMM cells were seeded at a density of 2 × 10^5^ cells/well in a 24-well plate and treated with 10 *μ*g/ml of DCR3 or IgG control in the presence of RANKL and M-CSF. After 6 hours of incubation, total RNA was isolated, and 1 *μ*g of total RNA was used to reverse transcribe cDNA. Mouse IL-1*α*, sIL-1ra, and icIL-1ra were detected by RT-PCR (a) and QPCR (b–d). (e) RAW264.7 cells were treated with 10 *μ*g/ml DCR3 or IgG in the presence of RANKL (50 ng/ml) stimulation for 6, 24, or 48 hours. Cell extracts were analyzed by immunoblotting assay. Equal amounts of protein were loaded in each lane as demonstrated by the level of GAPDH. (f) Supernatants at 24 or 48 hours were analyzed by IL-1*α* ELISA. Cell extracts (g) and supernatants (h) at 24 or 48 hours were analyzed by IL-1ra ELISA. A representative result of at least three independent experiments is shown (M: BMM cells+MCSF; RM: RANKL+MCSF; RMG: RANKL+MCSF+IgG; RMD: RANKL+MCSF+DCR3; N: RAW264.7 cells; R: RANKL; RG or R+G: RANKL+IgG; RD or R+D: RANKL+DCR3; ^∗∗∗^*P* < 0.001).

To clarify whether the presence of DCR3 in RANKL-induced osteoclast differentiation was involved in IL-1*α*/IL-1ra regulation, we tested the expression levels of IL-1*α* and IL-1ra RNA using RT-PCR and QPCR in RAW264.7 cells and BMMs. The results showed that IL-1*α* mRNA was elevated within 6 hours after RANKL plus DCR3 stimulation in BMMs (Figures 2(a) and 2(b)) and RAW264.7 cells (sFigures 2A and 2C). Furthermore, the mRNA of intracellular IL-1ra (icIL-1ra) and secretory IL-1ra (sIL-1ra) were also elevated at 6 hours after RANKL plus DCR3 stimulation in BMM (Figures 2(a), 2(c), and 2(d)) and RAW264.7 cells (sFigures 2B, 2D, and 2E). We further analyzed IL-1*α* and IL-1ra protein expression during osteoclast differentiation. Our results showed that the expression level of IL-1*α* significantly increased in the cell lysate at 6 hours in the early phase (Figure 2(e) and sFigure 3) but not in the supernatant (Figure 2(f)). The IL-1ra levels dramatically increased in the cells and supernatants in the DCR3-treated group at 24 and 48 hours (Figures 2(g), 2(h), and sFigure 3).

### 3.3. Effects of DCR3 on Reactive Oxygen Species and IL-1*α* Localization during Osteoclast Differentiation

**Figure 3:**
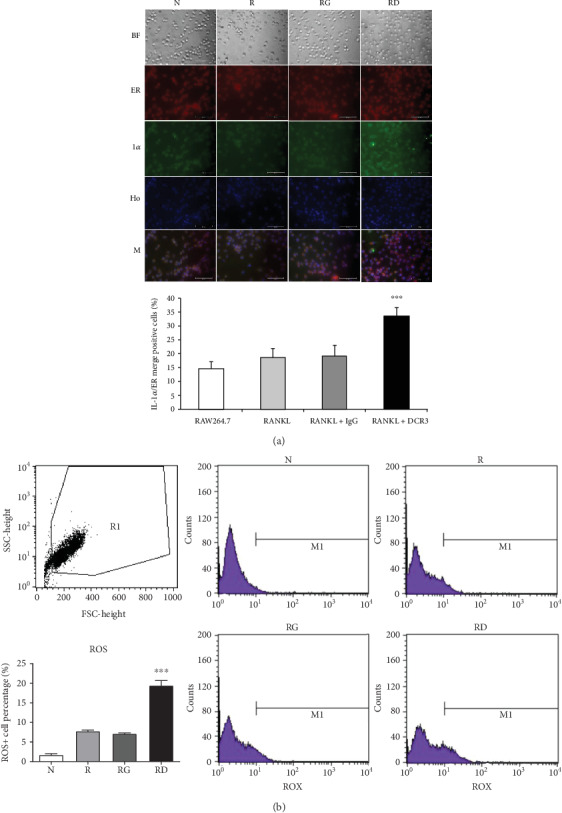
Effects of DCR3 on IL-1*α* protein localization and ROS expression in RANKL-induced osteoclast differentiation. RAW264.7 cells were treated with DCR3 or IgG in the presence of RANKL stimulation for 6 hours. After the time indicated, the cells were stained with an anti-IL-1*α* antibody (green), endoplasmic reticulum (red), and Hoechst dye (blue) or with a ROX detection kit. (a) The localization of IL-1*α* was compared in merged images for each group. (b) The ROS levels in each group were evaluated by flow cytometry. A representative result of at least three independent experiments is shown (BF: bright field; ER: endoplasmic reticulum staining; 1*α*: IL-1*α* staining; Ho: nuclear staining; M: merge image of ER, 1*α*, and Ho; N: RAW264.7 cells; R: RANKL; RG: RANKL+IgG; RD: RANKL+DCR3; ^∗∗∗^*P* < 0.001).

In previous studies, IL-1*α* has been shown to be upregulated in ER-stressed macrophages and localized to the nucleus in apoptotic cells [31, 32]. To further clarify whether IL-1*α* was induced by DCR3 in ER-stressed apoptotic cells during osteoclastogenesis, we used an immunofluorescence stain to detect the level and distribution of IL-1*α* in each group 6 hours after DCR3 treatment. Our results showed that IL-1*α* accumulated in the endoplasmic reticulum of cells concurrently treated with RANKL and DCR3 (Figure 3(a)). Moreover, previous studies have shown that IL-1*α* and IL-1ra are markers of sterile inflammation in hypoxia/ROS-induced cell death [33, 34]. To understand whether DCR3 was involved in hypoxia/ROS-induced cell death in RANKL-induced osteoclast differentiation, we tested the expression levels of ROS in RAW264.7 cells. Interestingly, the results showed that the ROS levels were significantly increased by two-fold in the DCR3-treated group compared to the levels in groups treated with RANKL or with RANKL plus an IgG control (Figure 3(b)).

### 3.4. Effects of DCR3 on the Mechanisms of Apoptosis during Osteoclast Differentiation

**Figure 4:**
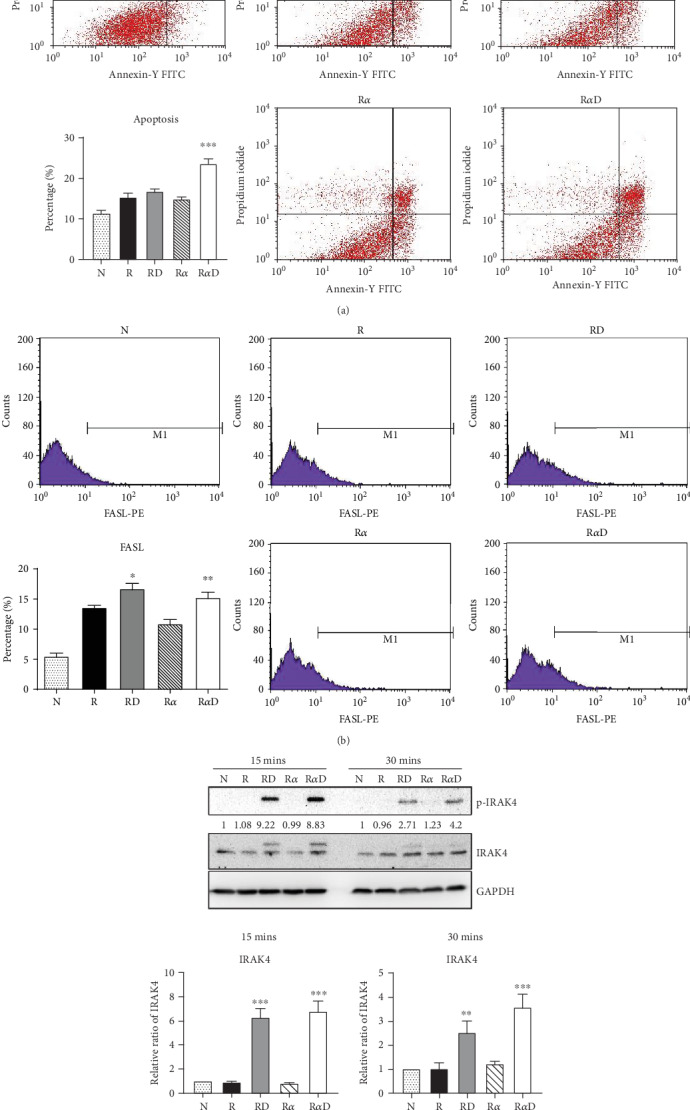
Effects of DCR3 on cell apoptosis and interleukin-1-associated kinase activation in osteoclasts. RAW264.7 cells were treated for 48 hours with or without 10 *μ*g/ml of DCR3 in the presence of RANKL or RANKL plus IL-1*α*. After incubation, cells were harvested for staining with (a) Annexin V and PI to evaluate the percentage of cell apoptosis or with (b) anti-Fas ligand to evaluate death ligand expression. (c) RAW264.7 cells were serum-starved overnight and treated with DCR3 in the presence of RANKL or RANKL plus IL-1*α* for 15 or 30 minutes. Cell extracts were analyzed by western blot analysis using antibodies specifically directed against the phosphorylated forms of IRAK4, compared to data obtained with antibodies directed against the unphosphorylated states of the kinases. Equal amounts of protein were loaded in each lane as demonstrated by the level of GAPDH. The expression ratio of phosphorylated/nonphosphorylated IRAK4 was quantified and normalized to the GAPDH. A representative result of at least three independent experiments is shown (N: RAW264.7 cells; R: RANKL; R*α*: RANKL+IL-1*α*; RD: RANKL+DCR3; R*α*D: RANKL+IL-1*α*+DCR3; ^∗^*P* < 0.05, ^∗∗^*P* < 0.01, and ^∗∗∗^*P* < 0.001).

Our previous work found that DCR3 enhances cell apoptosis in preosteoclasts by inducing Fas ligand expression. Here, we found that this phenomenon also occurred under IL-1*α* treatment concurrent with DCR3 treatment during RANKL-induced osteoclast differentiation (Figures 4(a) and 4(b)). Importantly, previous studies have reported that IRAK4 activation is a crucial process in inflammatory arthritis and participates in the Fas/FasL system induction of cytokine production and apoptosis in macrophages [35, 36]. Based on previous findings, we further investigated whether DCR3 was involved in the Fas/FasL induction of IRAK4 kinase activation during osteoclastogenesis in RAW264.7 cells. Surprisingly, the results indicated that the IRAK4 signal in RANKL-induced osteoclastogenesis was activated by DCR3 (Figure 4(c)).

## 4. Discussion

The present study is the first publication to find that DCR3 suppresses RANKL- plus IL-1*α*-induced osteoclastogenesis. The inhibitory effect of DCR3 was involved in the hypoxia/ROS- and IL-1*α*/IL-1ra-induced cell death signalling pathway. The first study of DCR3 in osteoclastogenesis was published by Yang et al., and they found that DCR3 itself, without RANKL stimulation, induces osteoclast formation, bypassing the NF-*κ*B signalling pathway, and induces TNF*α* expression [29]. However, another study by Tai et al. found that macrophage-specific CD68 promoter-driven DCR3 overexpression in transgenic mice strongly induces tumour-associated macrophages and IL-1ra and IL-10 expression; reduces proinflammatory cytokines (TNF*α* and IL-6); and does not affect MMP2, MMP9, or bone development in mice [27]. The controversial results concerning the role of DCR3 in macrophage differentiation and the expression of cytokines, such as TNF*α* in vitro and in vivo, raised our curiosity. Hence, we repeated the same experiment in vitro by using the DCR3 recombinant protein and full-length gene transient transfection. We found that DCR3 suppressed osteoclast formation in vitro and attenuated collagen-induced arthritis in a mouse model in vivo [25, 26]. In addition, the suppressive effect of DCR3 on osteoclastogenesis was through Fas ligand expression and intrinsic mitochondria-induced cell apoptosis [26]. However, there are no studies discussing the regulatory function of DCR3 on IL-1*α*/IL-1ra in osteoclastogenesis.

The IL-1*α* cytokine has complicated and multiregulatory functions in different cell types. IL-1*α* and IL-1ra counterbalance each other in keratinocytes. In osteoclasts, IL-1*α* activates several signalling cascades, including the Akt, ERK, JNK, and NF-*κ*B pathways [7]. IL-1*α* also acts in an autocrine manner in osteoclast formation and maintains osteoclast survival [37, 38]. Downregulation of IL-1*α*/*β* by knockout mice enhances bone mass [39]. Moreover, sterile inflammation proceeds via the IL-1*α* hypoxia/ROS pathway and regulates cell death [33, 34]. However, in vivo cell death for tissue repair and the acute monocyte response to cell death are much less dependent on the IL-1R-MyD88 pathway [40]. Hence, the biological function of IL-1*α* in monocyte/macrophage lineage cells may benefit cell survival and maintain tissue-specific macrophage functions, such as osteoclast processing. In addition, the cell death pathway of the IL-1*α*-induced IL-1R-MyD88 pathway may be independent in macrophages or osteoclasts. In our study, we found that DCR3 activated endogenous osteoclast IL-1*α* expression and subsequently induced IL-1ra expression like the counterregulated function in keratinocytes. Moreover, two isoforms of IL-1ra have been identified, and when intracellular IL-1ra is released during cell apoptosis it has a function equivalent to that of secreted IL-1ra [41, 42]. Our findings demonstrated that DCR3 induced abundant icIL-1ra expression in preosteoclasts. In addition, the dramatic increase of icIL-1ra was accompanied by the suppression of osteoclastogenesis and induction of preosteoclast cell death.

DCR3 and its ligands, FASLG, LIGHT, and TL1A, are highly expressed in many inflammatory osteolytic diseases and are involved in osteoclastogenesis and cell death [43–46]. Our previous study demonstrated that DCR3 increases apoptosis of osteoclasts by increasing the level of the major ligand FASLG. Moreover, by connecting the Fas ligand with mitochondrial cell death, ROS have been shown to be critical factors in regulating the Fas death system in macrophages [47]. Although many studies have reported that ROS plays a critical role in the maintenance of macrophage phagocytosis ability and osteoclast differentiation [5, 48], ROS still guided the intrinsic endoplasmic reticulum (ER) stress-induced apoptosis in macrophages [49]. Previous studies have also demonstrated that ROS regulates IL-1*α* and IL-1ra expression in hypoxia-induced cell death [33, 34]. The IRAK4 kinase also participates in the Fas/FasL system and mediated apoptosis in macrophages [38]. Here, we found that DCR3 enhanced the ROS levels as well as IL-1*α* and IL-1ra expression during osteoclastogenesis. The mechanism of cell death was involved in linking DCR3 with the Fas/FasL system-induced activation of IRAK4. This function resorted to DCR3 because it might bond with its natural cell surface ligand, FasL, internalized into the macrophage, and it might stop the FasL-transduced osteoclastogenesis signalling and put macrophages forward to ROS-ER stress cell death. In addition, hypoxia-induced ROS have been reported to be involved in tumour formation [50]. In this regard, DCR3 might suppress osteoclast formation on the one hand, but could promote tumour-like osteolytic disease formation, such as rheumatoid arthritis, on the other.

In conclusion, we provide the first evidence that DCR3 exhibits inhibitory effects on RANKL- plus IL-1*α*-stimulated osteoclastogenesis as well as on pit formation. The molecular mechanisms of this inhibition involve intracellular ROS accumulation, IRAK4 kinase activation, and ROS induction of Fas ligand, IL-1*α*, and icIL-1ra expression. The blockage of RANKL- plus IL-1*α*-stimulated osteoclastogenesis by DCR3 leads to preosteoclast cell death. In summary, our study may be used to determine the possible molecular mechanisms of DCR3 in osteoclastogenesis and may reveal DCR3 as a potential therapeutic agent that is beneficial for the treatment of inflammatory bone disorder diseases, such as gingivitis, which are involved in IL-1*α*/IL-1ra imbalances.

## Data Availability

The data used to support the findings of this study are available from the corresponding author upon request.

## Supplementary Materials

Supplementary Materials sFigure 1: effects of DCR3 on RANKL- plus IL-1*α*-induced osteoclast differentiation in BMMs. sFigure 2: effects of DCR3 on IL-1*α* and IL-1ra mRNA regulation in RANKL-induced osteoclast differentiation. sFigure 3: effects of DCR3 on IL-1*α* and IL-1ra protein expression on RANKL-induced osteoclast differentiation. Supplementary Table 1: list of murine PCR primers.
