# Supplementary material for: Decoy Receptor 3 Promotes Preosteoclast Cell Death via Reactive Oxygen Species-Induced Fas Ligand Expression and the IL-1α/IL-1 Receptor Antagonist Pathway
Source: Mediators Inflamm. 2020 Jun 10;2020:1237281. doi: 10.1155/2020/1237281 (PMC7303756; doi:10.1155/2020/1237281)
Supplement: Supplementary Materials — sFigure 1: effects of DCR3 on RANKL- plus IL-1α-induced osteoclast differentiation in BMMs. sFigure 2: effects of DCR3 on IL-1α and IL-1ra mRNA regulation in RANKL-induced osteoclast differentiation. sFigure 3: effects of DCR3 on IL-1α and IL-1ra protein expression on RANKL-induced osteoclast differentiation. Supplementary Table 1: list of murine PCR primers. [file 1237281.f1.pdf]

Supplementary figures

DCR3 affects osteoclast differentiation in BMMs

Figure 1

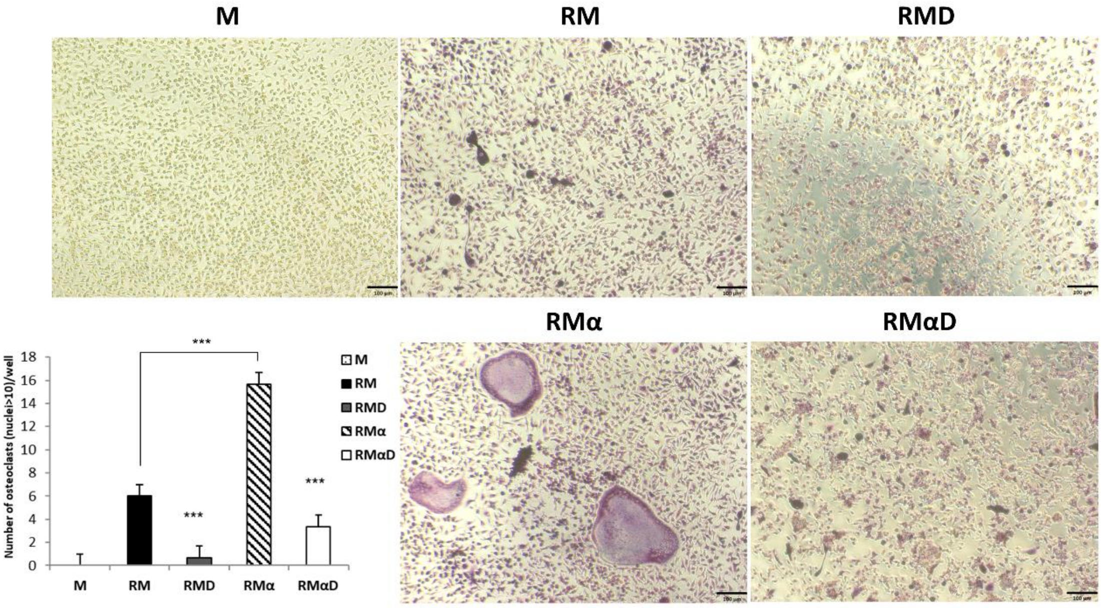

sFig. 1 Effects of DCR3 on RANKL plus IL-1α induced osteoclast differentiation in BMMs. BMMs were treated with DCR3 in the presence of RANKL (50 ng/ml) and M-CSF (30 ng/ml) or RANKL, IL-1α, and M-CSF for 5 days. After incubation, the cells were fixed and stained for TRAP and TRAP^{+} multinucleated cells containing more than five nuclei in RAW264.7 or ten nuclei in BMMs were counted as multinucleated osteoclasts. (M: BMM cells + MCSF; RM: RANKL + MCSF; RMD: RANKL + MCSF + DCR3; RMα: RANKL + MCSF + IL-1α; RMαD: RANKL + MCSF + IL-1α + DCR3) The data represent the means ± S.D. of more than three cultures. (***P < 0.001)

mRNA expression pattern of IL-1α, icIL-1ra, and sIL-1ra in DCR3 treated OC

Figure 2

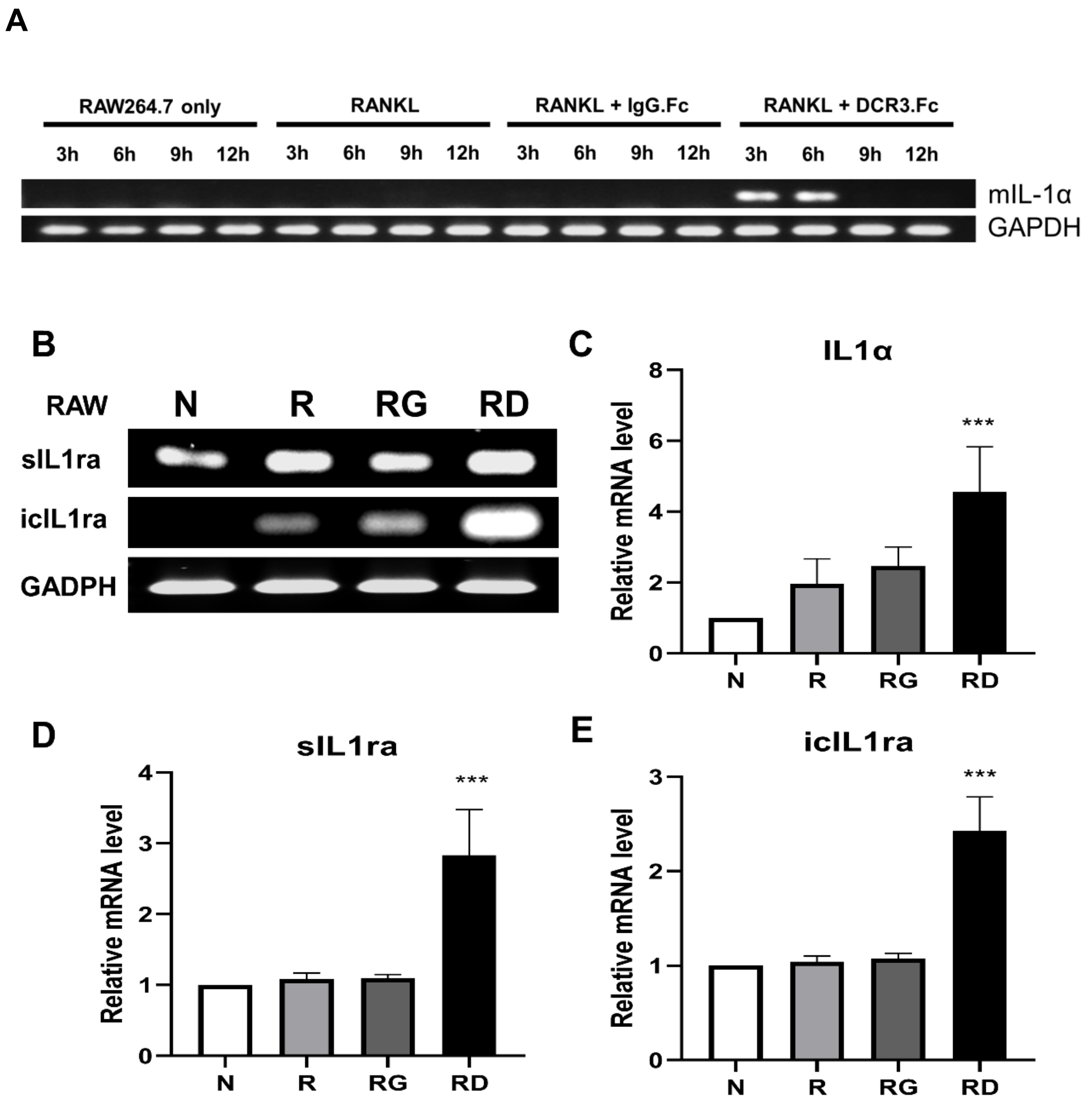

sFig. 2 Effects of DCR3 on IL-1α and IL-1ra mRNA regulation in RANKL-induced osteoclast differentiation. RAW 264.7 cells were seeded in density of $2x10^5$ cells/well at 24 well plate and treated with 10 μg/ml of DCR3 or IgG control in the presence of RANKL. After 3, 6, 9, and 12 hours treating with 10 μg/ml DCR3 or IgG control in the present of RANKL, total RNA was isolated and 1 μg of total RNA was used to transcribe cDNA. Mouse specific IL-1α, sIL-1ra, and icIL-1ra were detected by RT-PCR (A, B). According to the peak expression of IL-1α at 6 hours, IL-1α, sIL-1ra, and icIL-1ra were detected and quantified at 6 hours by QPCR.

A representative result of at least three independent experiments is shown. (N: RAW264.7 cells; R: RANKL; RG: RANKL + IgG; RD: RANKL + DCR3; ***$P < 0.001$)

Quantification of IL-1α and IL-1ra protein expression in DCR3 treated OC

Figure 3

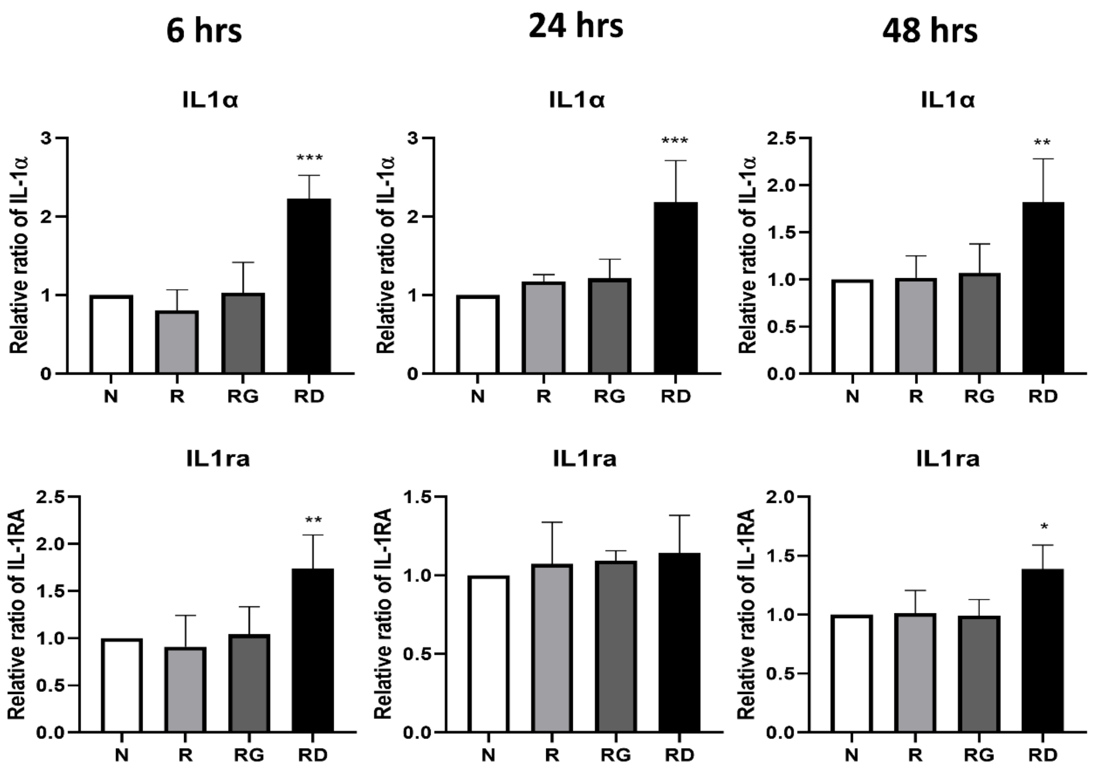

sFig. 3 Effects of DCR3 on IL-1α and IL-1ra protein expression on RANKL-induced osteoclast differentiation.

RAW264.7 cells were treated with 10 μg/ml DCR3 or IgG in the presence of RANKL (50 ng/ml) stimulation for 6, 24 or 48 hours. Cell extracts were analysed by immunoblotting assay. Equal amounts of protein were loaded in each lane as demonstrated by the level of GAPDH. A representative result of at least three independent experiments is shown. (N: RAW264.7 cells; R: RANKL; RG: RANKL + IgG; RD: RANKL + DCR3; *P < 0.05; **P < 0.01; ***P < 0.001)

List of murine PCR primers

Supplementary table

Table 1

Genes	Nucleotide sequences
IL-1α	5'- CGCTTGAGTCGGCAAAGAAA -3' (forward)
	5'- CTTCCCGTTGCTTGACGTTG -3' (reverse)
sIL-1ra	5'- CCTCGGGATGGAAATCTG -3' (forward)
	5'- CTGGTTGTTTCTCAGGTAAAAGG -3' (reverse)
icIL-1ra	5’- GCTCCTTTATACACAGCAAGTCTCT -3’ (forward)
	As antisense sIL-1ra
GAPDH	5'- GTGAGGCCGGTGCTGAGTATGT -3' (forward)
	5'- ACAGTCTTCTGGGTGGCAGTGAT -3' (reverse)
